# Resources, Production Scales and Time Required for Producing RNA Vaccines for the Global Pandemic Demand

**DOI:** 10.3390/vaccines9010003

**Published:** 2020-12-23

**Authors:** Zoltán Kis, Cleo Kontoravdi, Robin Shattock, Nilay Shah

**Affiliations:** 1 Centre for Process Systems Engineering, Department of Chemical Engineering, Faculty of Engineering, Imperial College London, London SW7 2AZ, UK; cleo.kontoravdi98@imperial.ac.uk (C.K.); n.shah@imperial.ac.uk (N.S.); 2 Department of Infectious Disease, Faculty of Medicine, Imperial College London, London W2 1PG, UK; r.shattock@imperial.ac.uk

**Keywords:** RNA vaccines, mRNA vaccines, saRNA vaccines, pandemic-response vaccine production, COVID-19, production process modelling, techno-economic analysis

## Abstract

To overcome pandemics, such as COVID-19, vaccines are urgently needed at very high volumes. Here we assess the techno-economic feasibility of producing RNA vaccines for the demand associated with a global vaccination campaign. Production process performance is assessed for three messenger RNA (mRNA) and one self-amplifying RNA (saRNA) vaccines, all currently under clinical development, as well as for a hypothetical next-generation saRNA vaccine. The impact of key process design and operation uncertainties on the performance of the production process was assessed. The RNA vaccine drug substance (DS) production rates, volumes and costs are mostly impacted by the RNA amount per vaccine dose and to a lesser extent by the scale and titre in the production process. The resources, production scale and speed required to meet global demand vary substantially in function of the RNA amount per dose. For lower dose saRNA vaccines, global demand can be met using a production process at a scale of below 10 L bioreactor working volume. Consequently, these small-scale processes require a low amount of resources to set up and operate. RNA DS production can be faster than fill-to-finish into multidose vials; hence the latter may constitute a bottleneck.

## 1. Introduction

The current COVID-19 pandemic has created an unprecedented demand for rapid and high-volume vaccine manufacturing. To meet this demand, several new platform technologies and conventional production approaches are being used [1]. Among these, the RNA vaccine platform technology holds great promise for gaining emergency use authorisation to facilitate immunisation against the SARS-CoV-2, which causes the COVID-19 respiratory disease [1,2]. However, this is a new technology, which means that there are considerable uncertainties regarding feasible manufacturing at high speed and scale. Here we report our findings from a production process and techno-economic modelling study in which we aim to answer the following questions related to the manufacturing productivity of the RNA vaccine platform: (1) How can we produce RNA vaccines and what are the key manufacturing uncertainties and challenges? (2) What production scales and resources are needed to manufacture RNA vaccines for immunising the world’s population against COVID-19? (3) How long will it take to manufacture vaccines to immunise the world’s population? (4) How to be better prepared for rapid-response manufacturing for future pandemics?

The answers to these questions also depend on the type of RNA vaccine because as of November 2020 there are four different RNA vaccine candidates in clinical development. Knowing that the RNA vaccine platform is a new technology, it is expected that this technology will further improve, for example by developing more effective formulations and by building more effective RNA constructs. As a result, we included a hypothetical next-generation RNA vaccine in this study whereby the RNA amount per dose may be further reduced without the use of modified nucleotides and assuming that only one dose is required to immunise a person. The details of the four existing RNA vaccines in clinical development together with the fifth, next generation, vaccine are listed in Table 1. As shown in Table 1, RNA vaccines fall into two broad categories, messenger RNA (mRNA) and self-amplifying RNA (saRNA) vaccines. In the latter type the RNA strands also encode for an alphaviral replication machinery, which enables amplification of the RNA upon delivery to the cytoplasm [3,4,5,6]. In addition, vaccines are also classified based on their developer and manufacturer, the amount of RNA per vaccine dose, the number of doses required to immunise a person, the type of uridine-5′-triphosphate (UTP) used for vaccine synthesis and their clinical development phase as of 20 November 2020.

## 2. Methods

### 2.1. Data Sources

Information regarding mRNA and saRNA vaccine production processes and costs was obtained from the scientific literature [4,5,6,15,16,17,18,19], patent databases [20,21,22,23,24], from GMP grade material suppliers, such as TriLink BioTechnologies Inc., San Diego, CA, USA and Roche Diagnostics GmbH, Mannheim, Germany. In addition, details regarding the configuration of the production process was obtained by active involvement of the authors in RNA vaccine production process development activities [25,26,27] and by discussing with contract manufacturing experts from TriLink BioTechnologies Inc., San Diego, CA, USA and Centre for Process Innovation Ltd., Darlington, UK. Information regarding mRNA DS amount per dose was obtained from clinical trial databases [28,29,30,31] and the scientific literature [11]. For saRNA vaccines the DS amount per dose was obtained from the clinical trial registry [14]. Additional production process data was obtained from the SuperPro Designer equipment, materials, utilities and cost databases [32]. The global demand for RNA vaccine DS was assumed at 8 billion doses.

### 2.2. Vaccine Production Process Modelling

The process flow diagram for the RNA vaccine production is shown in Figure S1 of the SI document. It was assumed that all five types of RNA vaccines included in this study are produced using production processes based on the same configuration of unit operations. The differences between these RNA production models were due to differences in the type and purchase price of UTP used, modified vs. wild-type, and the differences in the RNA amount per vaccine dose, as indicated in Table 1. The RNA drug substance production processes were modelled assuming production in single-use equipment.

RNA vaccine DS production related capital investment costs, operating costs, production volumes, production rates and cost per dose values were computed using SuperPro Designer Version 11, Build 2 from Intelligen, Inc (Scotch Plains, NJ, USA) assuming fed-batch operation mode. The SuperPro Designer bioprocess simulation tool is using built-in sets of algebraic and differential equations for the calculation of material and energy balances for each unit operation in the production process [32,33,34]. Based on the obtained results, SuperPro Designer sizes the equipment, calculates labour requirements, schedules the operations and procedures, and performs economic calculations both for capital expenses (CapEx) and operating expenses (OpEx) [32,33,34]. SuperPro Designer is linked to databases of chemicals, consumables, equipment and other resources, and these cost and other input values are also user-specifiable [32,33,34]. SuperPro Designer contains built-in algorithms for performing cost analysis, for user-adjustable cost settings and parameters see Table S1 in the SI document. The scale of the production processes was expressed based on the working volume of the production bioreactor and when processes were scaled up or down, the entire process was scaled proportionally to the scale of the bioreactor. It was assumed that the process can be scaled up to a limit of 30 L bioreactor working volume as scaling up above this limit was considered technologically not feasible. The production batches were scheduled for maximising equipment utilisation, thus maximising the number of production batches per year, however the production process was not de-bottlenecked.

To calculate the annual production amounts, the mass of RNA produced per year, expressed in g per year, computed using in SuperPro Designer, was divided by the amount of RNA per dose, expressed in µg per dose. To calculate the production cost per dose, the annual operating cost which also contained the annualised capital cost was divided by the total number of DS doses produced per year. The low, medium, and high scenarios presented in Section 2.2 were obtained by modifying the production titres by ±20% in the production bioreactor, relative to a baseline value of 5 g/L. Further details about techno-economic modelling are available in the SI document. The SuperPro Designer modelling files are available in a publicly accessible repository: https://github.com/ZKis-ZK/LNP-formulated-RNA-vaccine-drug-substance-production-cost-modelling

## 3. Results and Discussion

### 3.1. How Can We Produce RNA Vaccines and What Are the Key Manufacturing Uncertainties and Challenges?

The process flow diagram for RNA vaccine production is shown in Figure S1 of the SI document both for drug substance production (i.e., active ingredient production, bulk production, primary manufacturing) and drug product manufacturing (i.e., fill-to-finish or secondary manufacturing). The RNA drug substance production was modelled based on single-use equipment. The bottleneck for drug substance production is the formulation step, whereby the RNA is encapsulated into lipid nanoparticles (LNPs) using a controlled mixing process [8,35]. For this purpose, microfluidic mixing devices are commonly used to mix an aqueous stream containing the RNA with an ethanol stream containing the following four lipid components: phospholipids, polyethylene glycol (PEG) lipids, cholesterol and a proprietary ionisable lipid [35,36,37,38,39,40]. This bottleneck can be addressed by operating multiple microfluidic mixing devices in parallel or by using larger devices.

**Figure 1:**
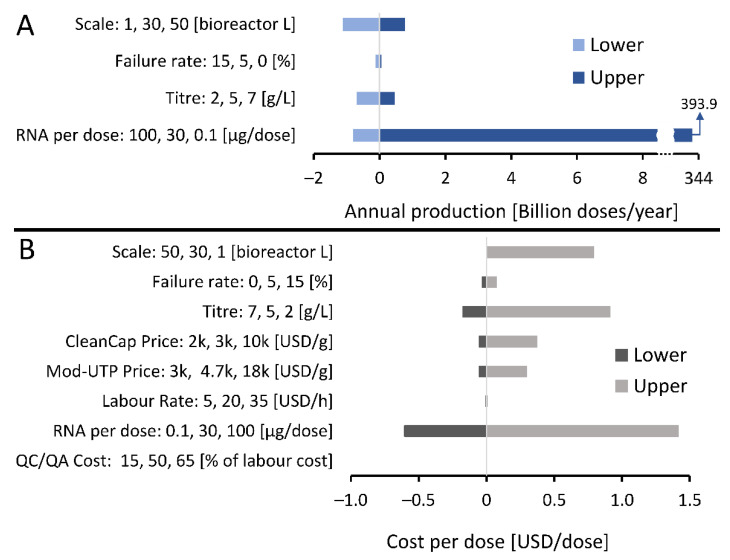
Sensitivity analysis showing RNA vaccine drug substance manufacturing uncertainties and their impact on annual production amounts and costs per dose. Input variables which are uncertain, their uncertainty ranges and units are shown on the vertical y-axis and the outputs of drug substance annual production amounts and costs per dose and shown on the horizontal x-axis. The zero values shown on the horizontal x-axes and the corresponding vertical line indicates a baseline scenario which describes an RNA vaccine production process with a 30 L bioreactor working volume scale, a final titre in the bioreactor of 5 g/L, 44% combined losses in the downstream purification and formulation steps, 30 µg of RNA per vaccine dose, a production process failure rate of 5%, 5′ cap analogue (CleanCap) purchase price of 3000 USD/g, 1-methyl-pseudouridine (Mod-UTP) purchase price of 4700 USD/g, basic labour rate of 20 USD/hour, quality control testing (QC/QA) cost of 50% of the labour costs, and 444–471 production batches completed per year. (**A**) The impact of uncertainties and their ranges listed on the vertical y-axis on the amount of doses worth of lipid nanoparticles (LNP) formulated RNA that can be produced annually shown on the horizontal x-axis. Results are shown relative to a baseline scenario at zero on the x-axis, as described above. (**B**) The impact of uncertainties and their ranges listed on the vertical y-axis on the production cost of the LNP formulated RNA drug substance per dose shown on the horizontal x-axis. Results are shown relative to a baseline scenario at zero on the x-axis.

Since the RNA vaccine drug substance platform is a new technology, there are uncertainties related to both the development and operation of large-scale production processes. These uncertainties can impact the performance of the manufacturing process in terms of production rates, volumes and costs. To assess this, the key uncertainties related to RNA vaccine drug substance production are listed on the y-axes in Figure 1 and their impact on the annual production amounts and on the cost of the drug substance (DS) per vaccine dose are shown on the x-axes in Figure 1. These variations in annual production amounts and production costs per dose are reported relative to a baseline scenario indicated with 0 on the x-axis and described in the figure legend.

As shown in Figure 1A, annual production amounts are most strongly impacted by the differences in RNA amount per vaccine dose, explained by the large variation in the range of possible RNA amounts per dose between 0.1 and 100 µg of RNA per dose. The annual production amount depends to a much lesser extent on changes in scale of the production process in the 1–50 L bioreactor working volume range, followed by the changes in production titre in the 2–7 g/L range. The scale of the production was expressed in L of bioreactor working volume. When the process was scaled up or down, the rest of the process was scaled proportionally with the bioreactor working volume. Changes in the failure rate of the production process in the 0–15 % range had a minimal impact on the RNA DS annual production amounts. The cost of the RNA DS per dose is impacted mostly by the amount of RNA per dose, followed by changes in production titres and in production scales, as shown in Figure 1B. The ranges in which the RNA amount per dose, scale, titre and failure rate varied were identical in Figure 1A,B as indicated on the y-axes of these figures. The RNA DS cost per dose was impacted to a smaller extent by the purchase price of the 5′ cap analogue (CleanCap, TriLink Biotechnologies, Inc. San Diego, CA, USA) and purchase price of the mod-UTP. Changes in the production process failure rate, labour costs and quality control testing (QC/QA) costs had minimal impact on the RNA DS cost per dose.

The production performance of the RNA vaccine production platform also depends on the scheduling, the time gaps between subsequent batches and the times required for the completion of the quality control tests. These aspects were not considered in this study.

The RNA vaccine drug substance production is a platform technology meaning that virtually any RNA sequence can be produced using the same unit operations of RNA synthesis, purification and formulation. The only component that needs to be changed in this process when switching to the production of a new vaccine is the DNA template based on which the RNA is synthesised using the T7 RNA Polymerase enzyme. The creation of a DNA template has been achieved within days after determining the genetic sequence of the SARS-CoV-2 virus [26,41,42]. The DNA template can be produced at large scale as plasmid DNA using well-established Escherichia coli (*E. coli*) fermentation-based processes [43,44,45] or by using enzymatic processes, such as the doggybone™ developed by Touchlight Genetics Ltd., U.K [46,47,48]. The circular plasmid DNA template needs to be linearised using a restriction enzyme [25,26]. The saRNA vaccines require a larger DNA template compared to mRNA vaccines, however these larger plasmids can be manufactured using both *E. coli* fermentation and the doggybone™ technology [43,44,45,46,47,48]. For the next-generation saRNA vaccines, potential areas of template DNA improvements include codon optimisation and optimisation of binding sites for the T7 RNA polymerase and for the alphaviral RNA replicase enzymes [3].

### 3.2. What Production Scales and Resources Are Needed to Manufacture RNA Vaccines for Immunising the World’s Population against COVID-19?

The answer to this question about resources and scale requirements depends on the type of the RNA vaccine to be used in global COVID-19 immunisation programs based on their differences in amounts per dose, the use of modified NTPs, and the number of doses required to immunise each person. Knowing that the global population is 7.8 billion and that most of the COVID-19 vaccines in late stage development require two doses per person, the total global demand for COVID-19 vaccine is 15.6 billion, if all the people in all age groups were to receive the vaccine. In this study, the market share of the LNP formulated RNA vaccine DS was assumed at 8 billion doses per year, knowing that other types of COVID-19 vaccines are also in development such as adenovirus vector, inactivated viral and recombinant protein vaccines [1]. The final number of RNA vaccine doses administered to the population would be less than 8 billion per year as losses are expected to occur in the fill-to-finish processes as well as during the distribution operations and at the administration locations.

**Figure 2:**
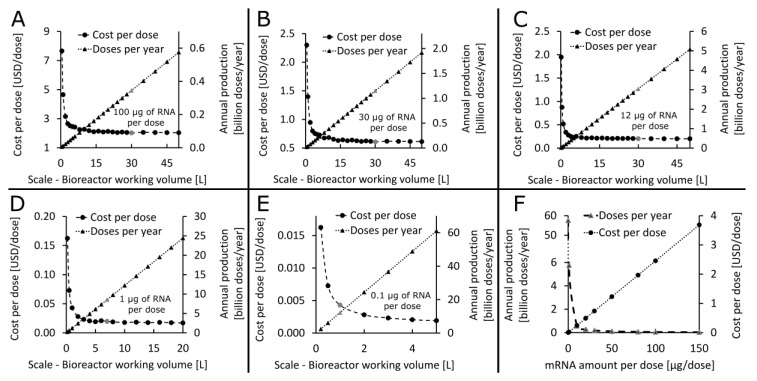
Determining the techno-economically feasible production scale for five vaccines with varying RNA amount per dose listed in Table 1. The entire process is scaled up proportionally and the scale is indicated by the bioreactor working volume. (**A**) Identifying the techno-economically feasible production scale for a 100 µg/dose RNA vaccine with modified UTPs. The annual production amounts and the drug substance production cost per dose is plotted in function of the scale of the production process. The scale identified as technologically feasible and economically viable corresponds to the 30 L bioreactor working volume and the corresponding key performance indicators (KPIs) of annual production and cost per dose values are shown by the grey dot and triangles, respectively. (**B**) Determining the suitable production scale for a 30 µg/dose RNA vaccine with modified UTPs. Plotting the annual production amounts and cost per dose in function of production scale identified the techno-economically feasible scale at 30 L bioreactor working volume, corresponding KPIs are indicated by the grey dot and triangle. (**C**) Determining the suitable production scale for a 12 µg/dose RNA vaccine with wild-type UTPs. Plotting the annual production amounts and cost per dose in function of production scale identified the techno-economically feasible scale at 30 L bioreactor working volume, corresponding KPIs are indicated by the grey dot and triangle. (**D**) Determining the suitable production scale for a 1 µg/dose RNA vaccine with wild-type UTPs. Plotting the annual production amounts and cost per dose in function of production scale identified the techno-economically feasible scale at 7 L bioreactor working volume, corresponding KPIs are indicated by the grey dot and triangle. (**E**) Determining the suitable production scale for a 0.1 µg/dose RNA vaccine with wild-type UTPs. Plotting the annual production amounts and cost per dose in function of production scale identified the techno-economically feasible scale at 1 L bioreactor working volume, corresponding KPIs are indicated by the grey dot and grey triangle. (**F**) The impact of the RNA amount per vaccine dose on the annual production amounts and cost per dose.

After the yearly demand was estimated, the production scales that are technologically feasible and economically viable were determined. Knowing that this is a new technology which has not been scaled up before, it was assumed that the production process can be scaled up to scales corresponding to 30 L bioreactor working volume. In cases when production at this scale is not sufficient for meeting the yearly demand, production can be scaled out. Therefore, the technologically feasible and economically viable scales have been determined for the four RNA vaccines in clinical development and the one additional next-generational vaccine, cf. Table 1, based on the assumed 30 L scalability limit. The results of this production process scaling assessment are shown in Figure 2. For the vaccines with 100, 30, and 12 µg of mRNA per dose, the production needs to be scaled up to the 30 L feasibility limit assumed in this study, achieving the annual production amounts shown by the grey triangles and right y-axis in Figure 2A–C. To meet the global annual demand of 8 billion doses, several facilities would be required at this scale of 30 L bioreactor working volume for these higher RNA dose vaccines. For the vaccine with 1 µg of saRNA per dose, a single production process at the 7 L bioreactor working volume scale can produce more than 8 billion DS doses per year, whereas for a vaccine with 0.1 µg of saRNA per dose this annual demand can be met using a process at the 1 L bioreactor working volume scale, as shown by the grey triangles and right y-axis in Figure 2D,E. The cost per dose decreases nonlinearly as the scale increases as shown in Figure 2A–E. The annual production amount varies linearly as a function of the production scale, cf. Figure 2A–E. On the other hand, the RNA amount per dose shows a multiplicative inverse (i.e., reciprocal or 1/x) relation with the annual production amount and a linear relation with cost per dose, cf. Figure 2F for a vaccine with modified UTPs. The breakdown of operating cost and cost per dose components for LNP formulated DS production is shown in Figure S2. The major cost component for all RNA vaccines in clinical development for COVID-19 as of November 2020 is the material costs and the second highest cost component is the cost of consumables. For the next-generation RNA vaccines the cost of consumables is expected to become predominant due to the very low saRNA amount per dose which requires less materials to produce. The high consumable cost is due to production in single-use equipment. Within the materials the CleanCap reagent (TriLink Biotechnologies, Inc. San Diego, CA, USA) is the major cost component for all five types of RNA vaccines.

It is worth noting that the production cost per dose presented here is only a fraction of the selling price of the vaccine, which also accounts for the fill-to-finish and distribution costs, marketing expenses, a profit margin and the cost of the R&D, preclinical development and clinical trial related expenses [26].

**Figure 3:**
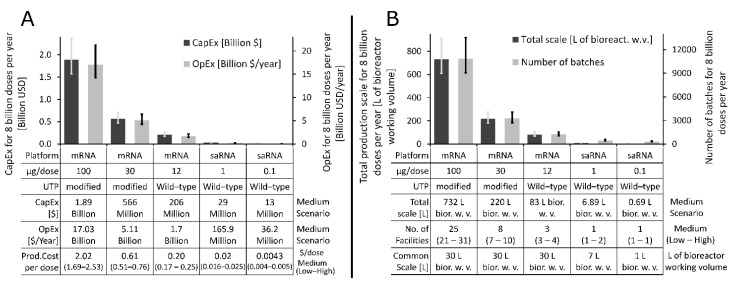
Resource and production scale requirements for producing 8 billion doses of LNP formulated RNA drug substance per year. Variations in production efficiencies were modelled by changing the production titre by −20% for the low scenario, leaving it at the 5/L baseline value for the medium scenario and increasing it by +20% for the high scenario. The bar charts represent the results for medium scenario and the error bars show the results for the low and high scenarios. (**A**) Total capital investment cost (CapEx) and annual operating cost (OpEx) required to produce 8 billion doses of RNA DS per annum for the five RNA vaccine types. The five vaccine types, their characteristics and cost-modelling results are shown in the table belonging to the x-axis. (**B**). Total production scales expressed in L of bioreactor working volume and the number of batches required to meet the RNA DS annual demand of 8 billion doses for the five RNA vaccine types. The five vaccine types and their common techno-economically feasible scales are shown in the table belonging to the x-axis. Additionally, this table also shows the total production scales required for meeting the 8 billion dose of DS annual demand and the number of facilities required to meet the same demand, assuming one production line at the techno-economically feasible scale per facility.

Once the most cost-effective production scale was identified for each of the five classes of RNA vaccines, we assessed the impact of variations in production efficiency on process performance and on the resources required to produce the 8 billion doses worth of LNP formulated RNA drug substance annually. Changes in production efficiency were modelled by changing the RNA titre in the production bioreactor by ±20% relative to the baseline value of 5 g·L^−1^. Thus, in the low scenario 4 g of RNA was obtained per L of bioreactor working volume, the medium scenario considered 5 g of RNA per L, and in the high scenario, 6 g of RNA was obtained per L of bioreactor working volume. The ±20% changes in titre are higher than what is expected for this process and this higher range can account for changes in the downstream purification efficiencies as well. The resources and production scales required to produce 8 billion doses of COVID-19 RNA vaccine DS annually based on the low, medium, and high scenarios for each of the five classes of RNA vaccines are shown in Figure 3 below. The medium scenario is shown by the bar charts and the low and high scenarios are illustrated with the low end and high end of the error bars, respectively. The annual operating expenditure (OpEx) and the total capital expenditure (CapEx) required to produce 8 billion doses of RNA vaccine DS annually are shown on the right and left y-axes of Figure 3A, respectively, for the five vaccine types. The table below the x-axis also shows the medium scenario values for DS production CapEx, OpEx, and cost per dose. The CapEx required to produce 8 billion doses of RNA DS per annum drops from the USD 1.57–2.36 billion range in the case of 100 µg/dose mRNA vaccine to USD 11–16.5 million range in the case of the 0.1 µg/dose saRNA vaccine. Similarly, The OpEx drops from the USD 14.2–21.3 billion per year range to USD 30.2–45.3 million per year range when moving from a 100 µg/dose mRNA vaccine to a 0.1 µg/dose saRNA vaccine.

The total combined production scale expressed in bioreactor working volume and the number of production batches needed to produce 8 billion doses of RNA DS per year are shown on the left and right y-axes of Figure 3B, respectively, for the scenarios described on the x-axis. The low, medium and high scenarios are again shown by the lower end of the error bars, the bar charts and the higher end of the error bars, respectively. The table belonging to the x-axis also lists the medium scenario for the total scale required for the annual production of 8 billion doses of RNA DS, as these values for the lower dosage vaccines are difficult to understand in the bar charts. The table belonging to the x-axis also shows the common scale at which the production processes can be technologically feasibly implemented for the five vaccine types.

The total scale required to produce 8 billion doses of RNA DS per year decreases from the 610–915 L range in case of the 100 µg/dose mRNA vaccine to 0.57–0.86 L range in case of the 0.1 µg/dose saRNA vaccine, as shown on the left y-axis of Figure 3B and in the table below the x-axis of Figure 3B. The number of batches required to meet the yearly demand of 8 billion doses worth of RNA DS, assuming the feasible scales shown in the table below the x-axis, would decrease from the 9030–13,544 batches range for the 100 µg/dose mRNA vaccine to 271–407 batches range in case of the 0.1 µg/dose saRNA vaccine, as shown on the right y-axis of Figure 3B. In addition, the table below the horizontal x-axis in Figure 3B also shows the number of facilities required to produce 8 billion doses worth of RNA DS per year assuming one production line per facility at the common scale which was previously identified as techno-economically feasible. Thus, for the 100 µg/dose mRNA vaccine 21–31 facilities are required to produce 8 billion doses worth of RNA DS per year, depending on the production efficiencies, as described above. These 21–31 facilities would house one 30 L scale process per facility. On the other hand, to produce 8 billion doses worth of RNA DS per year for the next generation vaccine, which is assumed to have 0.1 µg/dose saRNA per vaccine dose, one facility with a 1 L scale process would be sufficient. Overall, the scales required to produce the LNP formulated RNA vaccine DS for meeting the global demand are substantially smaller than the scales required to produce DS using conventional mammalian cell-based approaches [25,49].

If it becomes possible to achieve immunisation with a single dose of next generation RNA vaccines, the global vaccine demand could be more easily met and the vaccination programs could be carried out faster with less complexity.

### 3.3. How Long Will It Take to Manufacture Vaccines to Immunise the World’s Population?

**Figure 4:**
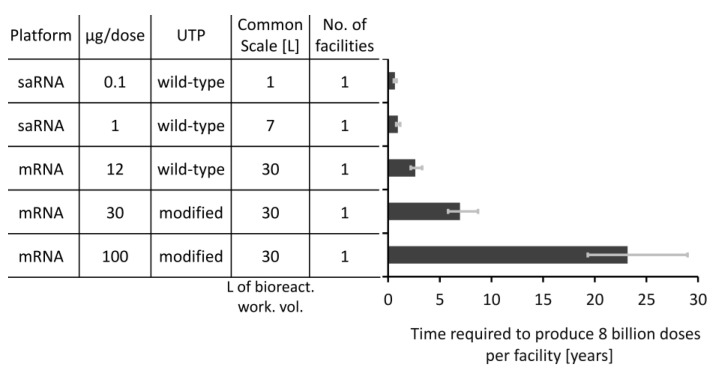
The time required to produced 8 billion doses worth of LNP formulated drug substance for the five RNA vaccine types. The times required per facility are shown on the x-axis and the five vaccine types together with their key features are shown in the table belonging to the y-axis. For this comparison it was assumed that a single facility housing a single production line at the techno-economically feasible scale is used. The error bars represent the low and high productivity scenarios which were obtained by adjusting the titre by ±20% from its baseline value of 5 g/L which corresponds to the medium scenario shown by the vertical bars of the chart.

The rate of vaccine manufacturing is also crucial, especially in a pandemic response situation. To assess the rate at which these five types of RNA vaccines can be produced, we computed the time required to produce 8 billion doses of the LNP formulated RNA vaccine DS using a single facility with a single production line at the scale that was described as techno-economically feasible in Figure 2. The results of this production time comparison are shown in Figure 4, with the required time illustrated on the x-axis. The table belonging to the y-axis also lists the key features of these five vaccine types. To take into account uncertainties that can impact the performance of the production process, the RNA synthesis titre was varied by ±20% and this way low, medium and high scenarios were obtained by setting the titre of the RNA in the bioreactor to 4, 5, and 6 g/L, respectively. The time required to complete a batch changes slightly as a function of production scale and ranges from 41 h in case of the 1 L scale process (for the production of the 0.1 µg/dose saRNA vaccine) to 48.2 h in case of the 30 L scale process (for the production of the 100 µg/dose mRNA vaccine). Consequently, the maximum number of batches that can be produced per facility decreases from 471, in case of the 1 L process, to 444, in case of the 30 L process, when increasing the production scale.

The time required to produce 8 billion doses of vaccines varies substantially between the five vaccine types. The 0.1 µg/dose saRNA vaccine can be produced at volumes of 8 billion doses within 6.5 and 9.8 months using a single facility housing a 1 L bioreactor working volume scale production line. The time required to meet this demand can be further shortened if the production processes are scaled up or if multiple production lines or facilities are used. On the other extreme, it would take between 19 and 29 years to produce 8 billion doses of DS for the 100 µg/dose mRNA vaccine using a single production line at the 30 L bioreactor working volume scale. This would be infeasible for a pandemic-response scenario and production would need to be scaled out or alternative vaccine production technologies would need to be employed. Therefore, Figure 4 together with Figure 2F illustrate that the amount of RNA per vaccine dose has a large impact on the rate and amount of RNA vaccine DS production. The vaccines that contain a low RNA amount per dose can be produced substantially faster if the processes are scaled up to the technologically feasible limit. For example, 8 billion vaccine DS doses of the 1 µg/dose vaccine can be produced in 2.6 months at the 30 L bioreactor working volume scale. The same 8 billion doses of the next-generational vaccine with 0.1 µg/dose can be produced in only 8 days using a process at the 30 L bioreactor working volume scale. When the DS is produced at such high rates and volumes, the fill-to-finish processes may not be able to match these production rates.

Pfizer Inc. (New York, NY, USA) and BioNTech SE (Mainz, Germany) projects that by the end of 2021 it will produce a total of 1.3 billion doses of its BNT162b2 vaccine, which has 30 µg of mRNA per dose [50]. The production of the BNT162b2 vaccine will take place in several facilities in parallel both in the US and in Europe. This 1.3 billion annual production amount value is in line with the productivity of a process at the 30 L scale as shown in Figure 2B. However, the actual production setup at Pfizer and BioNTech could be different, for example consisting of several, potentially smaller, production lines. Operational differences might also be caused by differences in scheduling, for example due to additional quality control testing time requirements. Moreover, producing 1.3 billion doses of the Pfizer and BioNTech vaccine per year also requires fill-to-finish capacity that could also pose challenges at this production scale and rate. Moderna Inc. (Cambridge, MA, USA) announced the production of a total of 125 million doses of its COVID-19 vaccine candidate in the first quarter of 2021—this vaccine contains 100 µg of mRNA per dose [51]. By the end of 2021 Moderna expects to produce between 500 million and 1 billion doses annually [52]. Additionally, Moderna has outsourced the manufacturing of its mRNA-1273 COVID-19 vaccine to Lonza in Basel, Switzerland, whereby four production lines are being developed to produce 100 million doses per year per production line [52]. This lower productivity of the Moderna process compared to the Pfizer and BioNTech process is in line with the higher amount of RNA per dose in the Moderna vaccine. However, the scale and number of production lines at these two vaccine developing and manufacturing industrial groups may differ from each other.

### 3.4. How to Be better Prepared for Rapid-Response Manufacturing for Future Pandemics?

It is worth noting that the analysis presented in this study focuses on RNA drug substance production and does not account for the resources and time required to fill the vaccines into vials. The fill-to-finish process can become a bottleneck as the RNA amount per dose decreases and a higher number of doses worth of drug substance (DS) is produced faster. In a pandemic response situation this fill-to-finish bottleneck would be worsened because the DS can be produced and stockpiled in parallel to clinical trials, with vials filled only once dosage has been confirmed based on clinical findings. Thus, there will be a backlog of DS for the fill-to-finish processes. In addition, vaccines DS produced using different technologies (e.g., viral vectored vaccines, inactivated viral vaccines, recombinant protein vaccines, etc.) may need to be filled using the same filling facilities. To address this fill-to-finish bottleneck, new technologies are being developed, such as filling 200 or 400 doses worth of DS into plastic bags [32]. Using this technology, if the vaccine formulation is thermostable and compatible, 400-dose bags can be filled at a rate of around 1.24 billion doses per month per filling line [53,54]. This is based on filling 116,250 pouches per day in three work shifts using a 15-needle INTACT™ Modular Filler from MEDInstill Development LLC, considering operation at 90% overall equipment effectiveness (OEE) [53,54].

There are also logistical challenges related to the distribution and administration of vaccines, which are aggravated by the urgency and scale required in pandemic response vaccination campaigns. The BNT162b2 vaccine developed by BioNTech and Pfizer requires distribution and storage at −70 °C and the mRNA-1273 vaccine developed by Moderna requires distribution and storage at −20 °C [2]. The low, and especially the ultra-low temperature storage at −70 °C, is costly and requires special freezers that may not be normally present at the distribution or vaccination centres. The distribution of such thermo-sensitive vaccines is even more challenging in low- and middle-income countries, where the required cold or ultracold chain infrastructure is not in place. Due to the high thermo-sensitivity and short shelf-life of these vaccines at higher temperatures, losses of vaccine doses could occur during distribution and administration to patients. Thus, overproduction of vaccines is likely to be required to make up for the losses. Some of these supply chain challenges could be tacked with implementing a distributed manufacturing approach with shortened distribution chains. These could become economically viable when using these low-scale, high-volume RNA vaccine production platform technologies. Developing more thermostable formulations would also simplify distribution logistics and reduce distribution costs.

To be in a better position for producing vaccines against future outbreaks, vaccine production surge capacity should be maintained, by having dedicated pandemic-response facilities established ahead of outbreaks. Once an outbreak occurs, these facilities could rapidly produce vaccine candidates and then vaccines to overcome the outbreaks. This would be relatively easily achieved using the RNA vaccine platform technology as the same production process offers the flexibility of producing a wide range of different vaccines and vaccine candidates. In addition, in the case of RNA vaccines that have low amounts per dose, high-volume and rapid production can be achieved using small scale production lines housed in small facilities. These production lines can also operate based on single-use equipment, which allows for fast switching to produce a different RNA vaccine against a different viral disease target. Therefore, when there is no outbreak, instead of being idle, these facilities could also produce other high-demand vaccines or vaccine candidates for clinical trials.

Moreover, these transformative vaccine production platform technologies can be combined with computational modelling tools to further accelerate both the development and production of vaccines against a wide range of pathogens, including currently unknown viral diseases [26]. The acceleration during development can be achieved by using a Quality by Design bioprocess modelling framework which incorporates disease-agnostic prior knowledge, production process understanding, expert knowledge, current experimental and clinical data [26]. Such a QbD model can process this data and run a large number of scenarios through an optimisation algorithm to identify the set of input variables and scenarios which lead to the most favourable outcomes in terms of product quality and quantity at the lowest cost and in the shortest time. This model can then also be adapted for automating and optimising the production process in real-time using model predictive control [26,55,56,57]. Furthermore, techno-economic analysis can be used to map out and guide the improvement of the performance of the entire production process by lowering costs, increasing production volumes and production rates, as also illustrated in this study.

## 4. Conclusions

In this computational modelling study, the techno-economic performance of the RNA vaccine production platform was evaluated for the COVID-19 RNA vaccine types that were in clinical development in November 2020 and for a next-generation saRNA vaccine that could be obtained by further improving this new technology. The RNA vaccine production process was presented and key uncertainties and variations both in the design and operation of the RNA drug substance production was presented. Moreover, the impact of these uncertainties and variations on the key performance indicators of the process was also evaluated, establishing that the RNA amount per vaccine dose has the highest impact both on the RNA drug substance annual production amounts and vaccine drug substance cost per dose, followed by the production scale and titre. Next the scalability requirements for RNA vaccine drug substance production were evaluated for the five types of vaccines, assuming a scalability limit corresponding to 30 L bioreactor working volume. If scaling above this limit is required a scaling out approach was assumed. The production scales and resources required to produce 8 billion doses of RNA drug substance for the global demand varied substantially among the five RNA vaccine types included in this study. This variation in production scales was due to the differences in RNA amount per vaccine dose, however these scales are still substantially smaller than in case of conventional vaccine manufacturing technologies. The RNA vaccine production scales required to meet the global demand would decrease from several hundreds of litres for vaccines with high amount of mRNA per dose to less than one litre bioreactor working volume for vaccines with low saRNA amount per dose. Consequently, the annual operating costs required to meet the global demand would drop from ≈17 billion USD/year in case of vaccines with 100 µg of mRNA per dose to ≈36.6 million USD/year in case of vaccines with 0.1 µg of saRNA amount per dose. Similarly, the time required to produce vaccines for a global pandemic demand would vary by several orders of magnitude between a high RNA dose vaccine and a low RNA dose vaccine. When producing the vaccine drug substance at such a high dose volume and rate, the fill-to-finish process could become a bottleneck. These platform technologies will enable faster vaccine development and production for overcoming future epidemics and pandemics, especially if surge manufacturing capacity is maintained. This is achievable at low costs when using these small-scale high throughput platform technologies in combination with single use equipment and digital tools to aid both vaccine development and production.

## Figures and Tables

**Table 1:** List of RNA vaccines considered in this study.

No.	Vaccine Type	Developer	Vaccine Name	RNA Per Dose (µg/Dose)	Probable Doses Per Person	Type of UTP *	Manufacturing Location	Clinical Development Phase	Ref.
1	mRNA	Moderna Inc. NIAID	mRNA-1273	100	2	Modified	USA, Spain, Switzerland,	Phase 3 clinical trial	[1,7,8,9]
2	mRNA	BioNTech SE; Pfizer Inc.	BNT162b2	30	2	Modified	USA, Germany, Belgium	Phase 3 clinical trial	[1,8,10,11,12]
3	mRNA	CureVac N.V.	CVnCoV	12	2	Wild-type	Germany	Phase 2 clinical trial	[1,8,13]
4	saRNA	Imperial College London	LNP-nCoVsaRNA	1	2	Wild-type	UK	Phase 1/2 clinical trial	[1,3,14]
5	saRNA	T.B.D. **	T.B.D. **	0.1	1	Wild-type	T.B.D. **	N.A.	

* The type of uridine-5′-triphosphate (UTP) used: modified means N1-methylpseudouridine-5′-triphosphate, and wild-type is nonmodified uridine-5′-triphosphate found in common mRNA. ** T.B.D.—to be determined. This assumes a next-generation saRNA vaccine that requires a lower amount per dose without the use of modified UTPs.

## Data Availability

The SuperPro Designer modelling files and data is available in a publicly accessible repository: https://github.com/ZKis-ZK/LNP-formulated-RNA-vaccine-drug-substance-production-cost-modelling.

## Supplementary Materials

The following are available online at https://www.mdpi.com/2076-393X/9/1/3/s1, Figure S1: Process flow diagram for RNA vaccine drug substance production (i.e., active ingredient production, bulk production or primary manufacturing) and drug product manufacturing (i.e., fill-to-finish or secondary manufacturing). Figure S2: Breakdown of annual operating costs and cost per dose for LNP-formulated RNA drug substance production based on the five RNA vaccine types. Table S1: Input parameters and assumptions used for techno-economic modelling in SuperPro Designer.

## References

[1] World Health Organization (2020). DRAFT Landscape of COVID-19 Candidate Vaccines. https://www.who.int/publications/m/item/draft-landscape-of-covid-19-candidate-vaccines.

[2] Dolgin E. (2020). COVID-19 Vaccines Poised for Launch, but Impact on Pandemic Unclear. Nat. Biotechnol..

[3] McKay P.F., Hu K., Blakney A.K., Samnuan K., Brown J.C., Penn R., Zhou J., Bouton C.R., Rogers P., Polra K. (2020). Self-amplifying RNA SARS-CoV-2 lipid nanoparticle vaccine candidate induces high neutralizing antibody titers in mice. Nat. Commun..

[4] Vogel A.B., Lambert L., Kinnear E., Busse D., Erbar S., Reuter K.C., Wicke L., Perkovic M., Beissert T., Haas H. (2018). Self-Amplifying RNA Vaccines Give Equivalent Protection against Influenza to mRNA Vaccines but at Much Lower Doses. Mol. Ther..

[5] Pardi N., Hogan M.J., Porter F.W., Weissman D. (2018). mRNA vaccines—A new era in vaccinology. Nat. Rev. Drug Discov..

[6] Geall A.J., Verma A., Otten G.R., Shaw C.A., Hekele A., Banerjee K., Cu Y., Beard C.W., Brito L.A., Krucker T. (2012). Nonviral delivery of self-amplifying RNA vaccines. Proc. Natl. Acad. Sci. USA.

[7] Jackson L.A., Anderson E.J., Rouphael N.G., Roberts P.C., Makhene M., Coler R.N., McCullough M.P., Chappell J.D., Denison M.R., Stevens L.J. (2020). An mRNA Vaccine against SARS-CoV-2—Preliminary Report. N. Engl. J. Med..

[8] Ye T., Zhong Z., García-Sastre A., Schotsaert M., De Geest B.G. (2020). Current Status of COVID-19 (Pre)Clinical Vaccine Development. Angew. Chem. Int. Ed..

[9] Servick K. (2020). This mysterious $2 billion biotech is revealing the secrets behind its new drugs and vaccines. Am. Assoc. Adv. Sci..

[10] Walsh E.E., Frenck R.W., Falsey A.R., Kitchin N., Absalon J., Gurtman A., Lockhart S., Neuzil K., Mulligan M.J., Bailey R. (2020). Safety and Immunogenicity of Two RNA-Based Covid-19 Vaccine Candidates. N. Engl. J. Med..

[11] Mulligan M.J., Lyke K.E., Kitchin N., Absalon J., Gurtman A., Lockhart S., Neuzil K., Raabe V., Bailey R., Swanson K.A. (2020). Phase I/II study of COVID-19 RNA vaccine BNT162b1 in adults. Nature.

[12] Sahin U., Muik A., Derhovanessian E., Vogler I., Kranz L.M., Vormehr M., Baum A., Pascal K., Quandt J., Maurus D. (2020). COVID-19 vaccine BNT162b1 elicits human antibody and TH1 T cell responses. Nature.

[13] Kremsner P., Mann P., Bosch J., Fendel R., Gabor J.J., Kreidenweiss A., Schunk M., Schindler C., Bosch J., Fendel R. (2020). Phase 1 Assessment of the Safety and Immunogenicity of an mRNA- Lipid Nanoparticle Vaccine Candidate Against SARS-CoV-2 in Human Volunteers. MEDRXIV.

[14] Fletcher J. (2020). Clinical Trial to Assess the Safety of a Coronavirus Vaccine in Healthy Men and Women. ISRCTN Regist..

[15] Schlake T., Thess A., Fotin-Mleczek M., Kallen K.-J. (2012). Developing mRNA-vaccine technologies. RNA Biol..

[16] Ljungberg K., Liljeström P. (2015). Self-replicating alphavirus RNA vaccines. Expert Rev. Vaccines.

[17] Reichmuth A.M., Oberli M.A., Jaklenec A., Langer R., Blankschtein D. (2016). mRNA vaccine delivery using lipid nanoparticles. Ther. Deliv..

[18] Brito L.A., Kommareddy S., Maione D., Uematsu Y., Giovani C., Berlanda Scorza F., Otten G.R., Yu D., Mandl C.W., Mason P.W. (2015). Chapter Seven-Self-Amplifying mRNA Vaccines. Adv. Genet..

[19] Hassett K.J., Benenato K.E., Jacquinet E., Lee A., Woods A., Yuzhakov O., Himansu S., Deterling J., Geilich B.M., Ketova T. (2019). Optimization of Lipid Nanoparticles for Intramuscular Administration of mRNA Vaccines. Mol. Ther. Nucleic Acids.

[20] Bancel S., Issa William J., Aunins John G., Chakraborty T. (2014). Manufacturing Methods for Production of RNA Transcripts. https://patentimages.storage.googleapis.com/7a/bb/8f/5ce58cdaa18a0d/US20160024547A1.pdf.

[21] Berlanda S.F., Wen Y., Geall A., Porter F. (2016). RNA Purification Methods. https://patents.google.com/patent/EP2970948A1/no.

[22] Funkner A., Dorner S., Sewing S., Kamm J., Broghammer N., Ketterer T., Mutzke T. (2016). A Method for Producing and Purifying RNA, Comprising at Least One Step of Tangential Flow Filtration. https://patentscope.wipo.int/search/en/detail.jsf?docId=WO2016193206.

[23] Wochner A., Roos T., Ketterer T. (2017). Methods and Means for Enhancing RNA Production. https://patents.google.com/patent/US20170114378A1/de.

[24] Heartlein M., Derosa F., Dias A., Karve S. (2014). Methods for Purification of Messenger RNA. https://patents.google.com/patent/DK2970955T3/en.

[25] Kis Z., Shattock R., Shah N., Kontoravdi C. (2019). Emerging Technologies for Low-Cost, Rapid Vaccine Manufacture. Biotechnol. J..

[26] Kis Z., Kontoravdi C., Dey A.K., Shattock R., Shah N. (2020). cRapid development and deployment of high-volume vaccines for pandemic response. J. Adv. Manuf. Process..

[27] Blakney A.K., McKay P.F., Yus B.I., Aldon Y., Shattock R.J. (2019). Inside out: Optimization of lipid nanoparticle formulations for exterior complexation and in vivo delivery of saRNA. Gene Ther..

[28] ModernaTX A Phase 2a, Randomized, Observer-Blind, Placebo Controlled, Dose-Confirmation Study to Evaluate the Safety, Reactogenicity, and Immunogenicity of mRNA-1273 SARS-COV-2 Vaccine in Adults Aged 18 Years and Older. https://clinicaltrials.gov/ct2/show/NCT04405076.

[29] ModernaTX A Phase 3, Randomized, Stratified, Observer-Blind, Placebo-Controlled Study to Evaluate the Efficacy, Safety, and Immunogenicity of mRNA-1273 SARS-CoV-2 Vaccine in Adults Aged 18 Years and Older. https://clinicaltrials.gov/ct2/show/NCT04470427.

[30] CureVac AG (2020). COVID-19: A Phase 2a, Partially Observer-blind, Multicenter, Controlled, Dose-confirmation Clinical Trial to Evaluate the Safety, Reactogenicity and Immunogenicity of the Investigational SARS-CoV-2 mRNA Vaccine CVnCoV in Adults >60 Years of Age and 18 to 60 Years of Age. https://clinicaltrials.gov/ct2/show/NCT04515147.

[31] CureVac AG (2020). A Phase 1, Partially Blind, Placebo-controlled, Dose-escalation, First-in-human, Clinical Trial to Evaluate the Safety, Reactogenicity and Immunogenicity After 1 and 2 Doses of the Investigational SARS-CoV-2 mRNA Vaccine CVnCoV Administered Intramuscularl. https://clinicaltrials.gov/ct2/show/NCT04449276.

[32] Petrides D. (2013). SuperPro Designer User Guide—A Comprehensive Simulation Tool for the Design, Retrofit & Evaluation of Specialty Chemical, Biochemical, Pharmaceutical, Consumer Product, Food, Agricultural, Mineral Processing, Packaging AND Water Purification, Wastewater. http://www.intelligen.com/downloads/SuperPro_ManualForPrinting_v10.pdf.

[33] Petrides D. (2015). Bioprocess Design and Economics. Bioseparations Science and Engineering.

[34] Petrides D., Carmichael D., Siletti C., Koulouris A. Biopharmaceutical Process Optimization with Simulation and Scheduling Tools. https://www.mdpi.com/2306-5354/1/4/154.

[35] Roces C.B., Lou G., Jain N., Abraham S., Thomas A., Halbert G.W., Perrie Y. (2020). Manufacturing considerations for the development of lipid nanoparticles using microfluidics. Pharmaceutics.

[36] Maier M.A., Jayaraman M., Matsuda S., Liu J., Barros S., Querbes W., Tam Y.K., Ansell S.M., Kumar V., Qin J. (2013). Biodegradable lipids enabling rapidly eliminated lipid nanoparticles for systemic delivery of RNAi therapeutics. Mol. Ther..

[37] Payne J.E., Chivukula P. (2014). Ionizable Cationic Lipid for Rna Delivery. https://patents.google.com/patent/WO2015074085A1/.

[38] Dammes N., Peer D. (2020). Paving the Road for RNA Therapeutics. Trends Pharmacol. Sci..

[39] Maeki M., Kimura N., Sato Y., Harashima H., Tokeshi M. (2018). Advances in microfluidics for lipid nanoparticles and extracellular vesicles and applications in drug delivery systems. Adv. Drug Deliv. Rev..

[40] Webb C., Forbes N., Roces C.B., Anderluzzi G., Lou G., Abraham S., Ingalls L., Marshall K., Leaver T.J., Watts J.A. (2020). Using microfluidics for scalable manufacturing of nanomedicines from bench to GMP: A case study using protein-loaded liposomes. Int. J. Pharm..

[41] O’Hare R., Lynch P. (2020). First Novel COVID-19 Vaccine Candidate Commences Animal Testing. https://www.univadis.co.uk/viewarticle/first-novel-covid-19-vaccine-candidate-commences-animal-testing-712575.

[42] Hodgson J. (2020). The Pandemic Pipeline. Nature Biotechnology. https://www.nature.com/articles/d41587-020-00005-z.

[43] Prazeres D.M.F., Ferreira G.N.M., Monteiro G.A., Cooney C.L., Cabral J.M.S. (1999). Large-scale production of pharmaceutical-grade plasmid DNA for gene therapy: Problems and bottlenecks. Trends Biotechnol..

[44] Schmeer M., Buchholz T., Schleef M. (2017). Plasmid DNA Manufacturing for Indirect and Direct Clinical Applications. Hum Gene Ther..

[45] Sahin U., Karikó K., Türeci Ö. (2014). mRNA-based therapeutics—Developing a new class of drugs. Nat Rev Drug Discov..

[46] Karda R., Counsell J.R., Karbowniczek K., Caproni L.J., Tite J.P., Waddington S.N. (2019). Production of lentiviral vectors using novel, enzymatically produced, linear DNA. Gene Ther..

[47] Karbowniczek K., Rothwell P., Extance J., Milsom S., Lukashchuk V., Bowes K., Smith D., Caproni L. (2017). Doggybone^TM^ DNA: an advanced platform for AAV production. Cell Gene Ther. Insights.

[48] Touchlight Genetics Ltd. (2020). Our Unique Synthetic DNA Vectors Advance Medicine and Manufacturing.

[49] Wen E.P., Ellis R.J., Pujar N.S. (2015). Vaccine Development and Manufacturing.

[50] Pfizer Inc. (2020). Pfizer and BioNTech Conclude Phase 3 Study of Covid-19 Vaccine Candidate, Meeting All Primary Efficacy Endpoints. https://www.pfizer.com/news/press-release/press-release-detail/pfizer-and-biontech-conclude-phase-3-study-covid-19-vaccine.

[51] Reuters Moderna to Supply up to 125 Million COVID-19 Vaccine Doses Globally in First Quarter. Reuters Health News.

[52] Reuters (2020). Lonza aims to make ingredients for 400 million doses of Moderna’s COVID vaccine annually. Reuters Healthc. Pharma.

[53] MEDInstill (2020). INTACTTM Modular Filler (IMF). http://www.medinstill.com/intact_modular_filler_imf.php.

[54] MEDInstill (2020). Email and Teleconference Correspondence with Experts from MEDInstill.

[55] Kroll P., Hofer A., Ulonska S., Kager J., Herwig C. (2017). Model-Based Methods in the Biopharmaceutical Process Lifecycle. Pharm. Res..

[56] Sommeregger W., Sissolak B., Kandra K., von Stosch M., Mayer M., Striedner G. (2017). Quality by control: Towards model predictive control of mammalian cell culture bioprocesses. Biotechnol. J..

[57] Mesbah A., Paulson J.A., Lakerveld R., Braatz R.D. (2017). Model Predictive Control of an Integrated Continuous Pharmaceutical Manufacturing Pilot Plant. Org. Process Res. Dev..

