# Correction: Kis, Z. et al. Resources, Production Scales and Time Required for Producing RNA Vaccines for the Global Pandemic Demand. *Vaccines* 2021, *9*, 3

**DOI:** 10.3390/vaccines9030205

**Published:** 2021-03-01

**Authors:** Zoltán Kis, Cleo Kontoravdi, Robin Shattock, Nilay Shah

**Affiliations:** 1 Centre for Process Systems Engineering, Department of Chemical Engineering, Faculty of Engineering, Imperial College London, London SW7 2AZ, UK; cleo.kontoravdi98@imperial.ac.uk (C.K.); n.shah@imperial.ac.uk (N.S.); 2 Department of Infectious Disease, Faculty of Medicine, Imperial College London, London W2 1PG, UK; r.shattock@imperial.ac.uk

The authors wish to make the following corrections to this paper [1].

In the original article, there was a mistake in the Supplementary Information file, lines 88–89, which mistakenly states that, “The purchase price of CleanCap 5’ capping analogues at GMP grade was received from the supplier, TriLink BioTechnologies Inc”. In addition and related to this, there was a mistake in the Methods section, Data Sources subsection of the article, according to which: “Information regarding mRNA and saRNA vaccine production processes and costs was obtained […] from GMP grade material suppliers, such as TriLink BioTechnologies Inc., San Diego, CA, USA.” This error was made due to a misunderstanding between the authors and TriLink BioTechnologies Inc. The corrected text appears below.

The purchase price of CleanCap AG or CleanCap AU stated in this publication were estimated by the authors and are not representative of actual pricing. TriLink BioTechnologies LLC (San Diego, CA, USA) did not supply CleanCap AG or CleanCap AU to Imperial College London at the purchase price estimated in this publication.

The authors apologize for any inconvenience caused and state that the scientific conclusions are unaffected. The original article has been updated.
