# Supplementary material for: Resources, Production Scales and Time Required for Producing RNA Vaccines for the Global Pandemic Demand
Source: Vaccines (Basel). 2020 Dec 23;9(1):3. doi: 10.3390/vaccines9010003 (PMC7824664; doi:10.3390/vaccines9010003)
Supplement: Supplementary file 1 [file vaccines-09-00003-s001.pdf]

Resources, production scales and time required for producing RNA vaccines for the global pandemic demand

Supplementary Informtion (SI) document

Zoltán Kis[1], Cleo Kontoravdi[1], Robin Shattock[2], Nilay Shah[1]

[1]Centre for Process Systems Engineering, Department of Chemical Engineering, Faculty of Engineering, Imperial College London, SW7 2AZ London, UK.

[2]Department of Infectious Disease, Faculty of Medicine, Imperial College London, W2 1PG London, UK.

1. Supplementary Figures

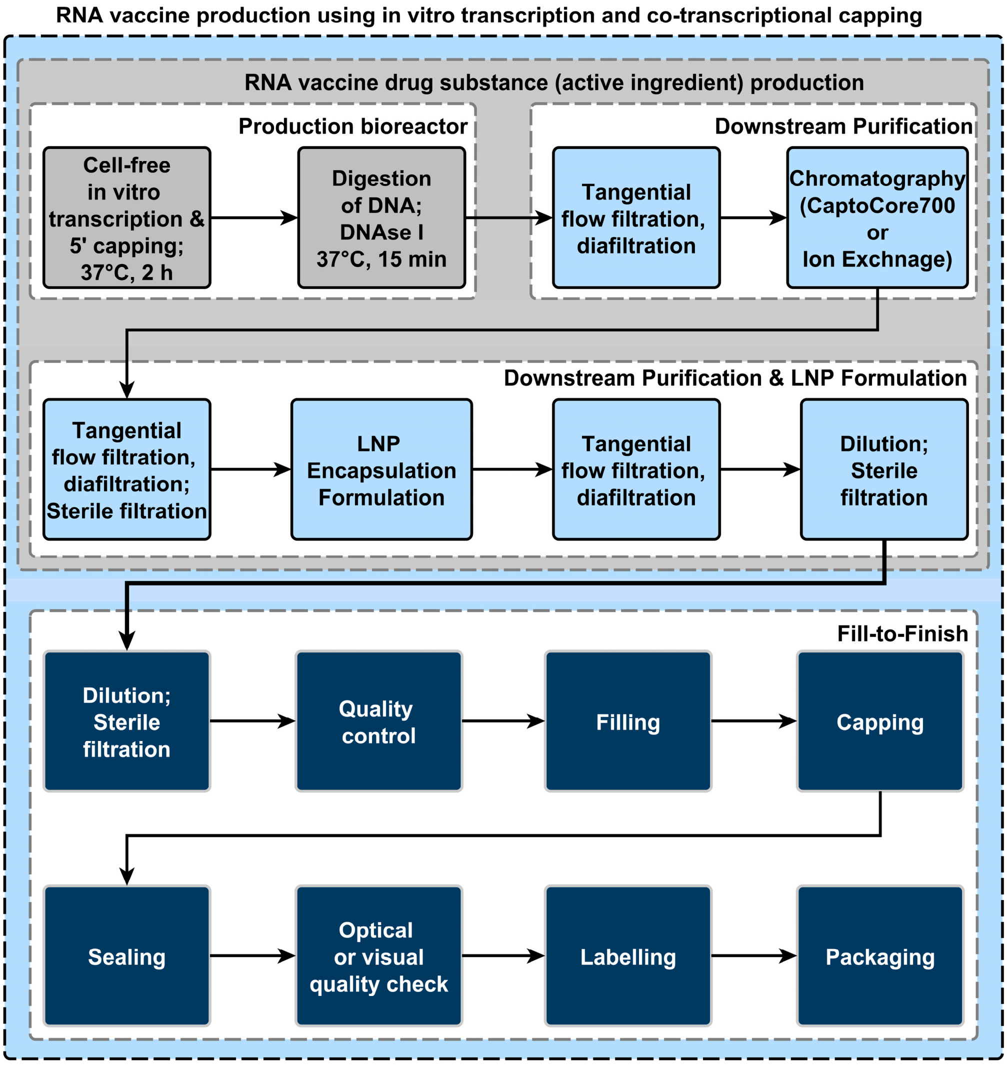

Figure S1. Process flow diagram for RNA vaccine drug substance production (aka. active ingredient production, bulk production or primary manufacturing) and drug product manufacturing (aka. fill-to-finish or secondary manufacturing). The drug substance is produced in the production bioreactor based on the *in vitro* transcription reaction using the T7 RNA polymerase enzyme, and 5' capping of the RNA is achieved co-transcriptionally using 5' cap analogues (needed to ensure antigen expression). Following RNA synthesis, the DNAse I enzyme is added to the bioreactor to digest the template DNA and then the reaction mix leaves the bioreactor and enters the downstream processing section. For downstream purification, tangential flow filtration (TFF) can be used to retain the RNA molecule by the filter and let the other components of the reaction mix flow through the TFF as these are smaller in size than the RNA molecule. Next, the retentate containing the RNA of interests is purified by a chromatography unit operation, such as CaptoCore 700 chromatography, ion exchange chromatography or hydroxyapatite chromatography, whereby the protein enzymes can be removed. Next, a second TFF step is carried out whereby the buffer is replaced for the formulation buffer and then the RNA solution is sterile filtered before entering the lipid nanoparticle (LNP) encapsulation unit operation which is the bottleneck for the RNA drug substance production (aka. primary manufacturing) section. Following the formulation step the LNP encapsulated RNA solution enters a third TFF for diafiltration then an optional dilution step is carried out followed by a sterile filtration operation. The sterile LNP-encapsulated RNA solution is then transferred to the fill-to-finish (aka. secondary manufacturing) section. There, an optional dilution step followed by sterile filtration can take place. Next, the formulated RNA solution undergoes quality control and is filled into vials or other containers. The vials are then capped, sealed, inspected using automated image processing, labelled and packaged into secondary and tertiary packaging. If blow-fill-seal [1,2] or the Intact™ Modular Filler [3,4] is used for fill-to-finish, than the filling, capping and sealing operation can be combined. The entire production process is independent of the RNA sequence, therefore in principle vaccines against virtually any disease can be produced using the same production process [4–10].

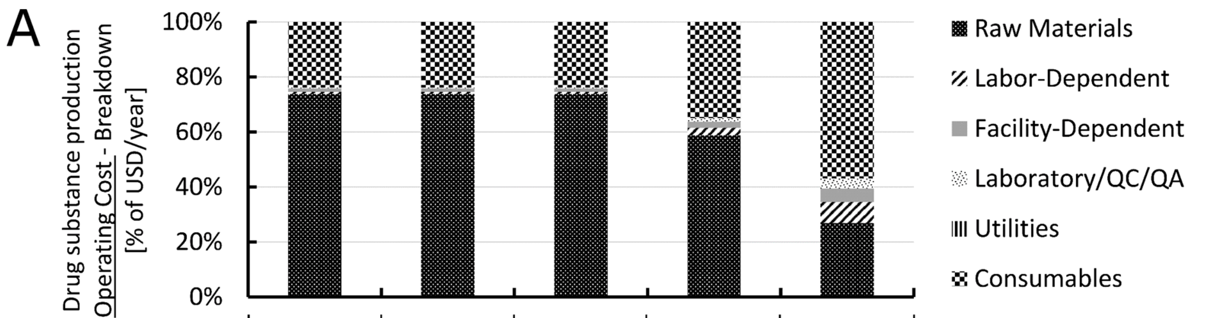

Platform	mRNA	mRNA	mRNA	saRNA	saRNA	
µg/dose	100	30	12	1	0.1	
UTP	modified	modified	Wild-type	Wild-type	Wild-type	
Common Scale [L]	30 L bior. w. v.	30 L bior. w. v.	30 L bior. w. v.	7 L bior. w. v.	1 L bior. w. v.	L of bioreactor working volume
OpEx [$/Year]	17.03 Billion	5.11 Billion	1.7 Billion	165.9 Million	36.2 Million	Total drug substance production Operating cost – Medium Scenario [$/dose]

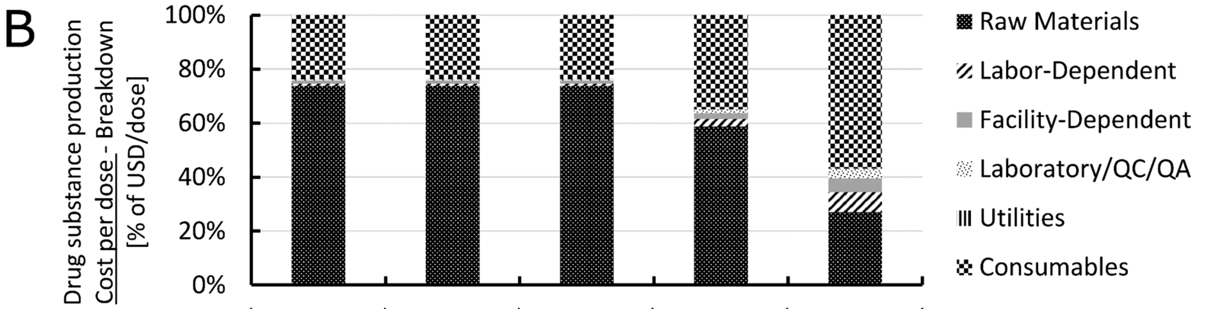

Platform	mRNA	mRNA	mRNA	saRNA	saRNA	
µg/dose	100	30	12	1	0.1	
UTP	modified	modified	Wild-type	Wild-type	Wild-type	
Common Scale [L]	30 L bior. w. v.	30 L bior. w. v.	30 L bior. w. v.	7 L bior. w. v.	1 L bior. w. v.	L of bioreactor working volume
Doses per year	345.085 Million	1.15 Billion	2.876 Billion	8.554 Billion	12.22 Billion	Doses produced per year per production line
Cost per dose	2.02	0.61	0.20	0.02	0.0043	Total drug substance production cost per dose – Medium Scenario [$/dose]

Figure S2. Breakdown of annual operating costs and cost per dose for LNP-formulated RNA drug substance production based on the five RNA vaccine types listed in Table 1. **A**. Share of operating cost (OpEx) components. The percentage of each OpEx component is shown on the y-axis and the five RNA types are shown on the x-axis. The table below the x-axis also indicates the total OpEx in USD per year for a single facility with a single production line at the common scale. **B**. Share of cost per dose components. The percentage of each cost per dose component is shown on the y-axis and the five RNA types are shown on the x-axis. The table below the x-axis also indicates the total cost per dose for LNP-formulated RNA drug substance production. The number of LNP-formulated drug substance doses produced per year are also shown for a facility at the common scale which is indicated in the table below the x-axis.

2. Supplementary methods

Techno-economic modelling

Drug substance production (aka. primary manufacturing) modelling as well as drug product manufacturing (aka. fill-to-finish, secondary manufacturing) modelling has been carried out using SuperPro Designer Version 11, Build 2 from Intelligen, Inc. The input parameters and assumptions for drug substance and drug product techno-economic modelling in SuperPro Designer are listed in **Table S1** below. Most of these parameters listed in Table S1 were kept at the default values from SuperPro Designer, as these default values are representative for biopharmaceutical production process and cost modelling. The Building Cost within the Direct Costs used for CapEx calculations was changed from the default value to 250 % of the total equipment purchase cost (TEPC), as this value is more representative of GMP production processes which have higher facility costs. Updating this Building Cost to 250 % of the TEPC was recommended by Demetri Petrides the from Intelligen, Inc who developed SuperPro Designer. The working capital cost period was decreased from the default value to 10 days because RNA vaccine production is faster compared to conventional cell-base biopharmaceutical production for which the default working capital cost period value was representative. The laboratory quality control (QC) and quality assurance (QA) costs were increased to 50% of the total labour costs (TLC) because this is a new technology and quality testing is likely to be more expensive compared to more established technologies. The impact of the QC/QA on the cost per dose was evaluated in Figure 1B and by changing the QC/QA costs between 15 – 65% of TLC the impact on the cost per dose was minimal. The time between consecutive batches (aka. cycle slack time) was set to 3 hours for production process models at the 30 L scale and to 2 hours for production process models at a lower scale. All production processes were modelled to operate 330 days per year. The number of campaigns per year was set to 1 in all the drug substance and drug product manufacturing models. The labour cost for drug substance production processes (operated in batch mode) was calculated using the detailed labour estimate, in function of the basic labour rate, benefits, operating supplies, supervision cost and administration cost. The labour cost for fill-to-finish processes (operated in continuous mode) was calculated using the lumped labour estimate. All other parameters shown in Table S1 were kept at the default values in SuperPro Designer.

Table S1. Input parameters and assumptions used for techno-economic modelling in SuperPro Designer.

Parameter use	Parameter class	Parameter name	Value	Unit
CapEx calculation	Direct Cost (DC)	Piping Cost	35	% of TEPC
		Instrumentation Cost	40	% of TEPC
		Insulation Cost	03	% of TEPC
		Electrical Facilities Cost	10	% of TEPC
		Buildings Cost	250	% of TEPC
		Yard Improvement Cost	15	% of TEPC
		Auxiliary Facilities Cost	40	% of TEPC
		Unlisted Equipment Purchase Cost (UEPC)	30	% of TEPC
		Unlisted Equipment Installation Cost	50	% of UEPC
	Indirect Cost (IC)	Engineering Cost	25	% of DC
		Construction Cost	35	% of DC
	Other Cost (OC)	Contractor's Fee	5	% of (IC + DC)
		Contingency	10	% of (IC + DC)

	Miscellaneous	Working Capital – to cover expenses for	10 mRNA & saRNA	days
		Start-up and Validation Costs	30	% of DFC
		Up front R&D	0	US$
		Up front royalties	0	US$
OpEx calculation	Facility dependent	Maintenance: equipment specific multipliers		
		Depreciation: contribution from each equipment's undepreciated purchase cost		
		Insurance	1	% of DFC
		Local taxes	2	% of DFC
		Factory expenses	5	% of DFC
	Labour	Basic operator labour rate (BOLR)	25	USD × hour^{-1}
		Benefits factor	40	% of BOLR
		Operating supplies factor	10	% of BOLR
		Supervision factor	20	% of BOLR
		Administration factor	60	% of BOLR
		Lumped operator labour rate	57.5	USD × hour^{-1}
		Adjusted basic operator labour rate*	57.5	USD × hour^{-1}
		Direct labour time utilization - batch	60	%
		Direct labour time utilization - continuous	70	%
	Lab, QC, QA	Laboratory, quality control, quality assurance	50	% TLC
	Utilities	Standard electricity	0.1	US$× (kW×h)$^{-1}$
		Chilled water	0.4	US$ × tonne^{-1}
		Cooled water	0.1	US$ × tonne^{-1}
		Steam	12	US$ × tonne^{-1}
	Miscellaneous	Fixed R&D	0	US$ × year^{-1}
		Variable R&D	0	US$ × g MP^{-1}
		On-going process validation	0	US$ × year^{-1}
		Other fixed	0	US$ × year^{-1}
		Other variable	0	US$ × g MP^{-1}
Overall economic evaluation	Time valuation	Construction period	20	months
		Start-up period	4	months
		Project lifetime	20	years
		Inflation	4	%
		NPV interest - Low	7	%
		NPV interest - Medium	9	%
		NPV interest - High	11	%
	Financing	Loan interest for DFC	9	%
		Loan interest for working capital	12	%
		Loan interest for up front R&D	12	%
		Loan interest for up front royalties	12	%
		Loan period for DFC	10	years
		Loan period for working capital	6	years
		Loan period for up front R&D	6	years
		Loan period for up front royalties	6	years
		DFC outlay for 1st year	30	% of DFC

		DFC outlay for 2nd year	40	% of DFC
		DFC outlay for 3rd year	30	% of DFC
		DFC outlay for 4th year	0	% of DFC
		DFC outlay for 5th year	0	% of DFC
		Straight line depreciation period	10	years
		Salvage value	5	% of DFC
	Production level	Operating capacity for each year	100	%
		Product failure rate	5	%
		Disposal cost	0	US$ × g MP^{-1}
	Miscellaneous	Income tax	40	%
		Fixed advertising and selling expenses	0	US$ × $year^{-1}$
		Variable advertising and selling expenses	0	US$ × g MP^{-1}
		Variable running royalty expenses	0	US$ × g MP^{-1}

Abbreviations used in Table S1: CapEx – capital expenditure; OpEx – operating expense; TEPC – total equipment purchase cost; UEPC – unlisted equipment purchase cost; DFC – direct fixed capital; DC – direct cost; IC – indirect cost; OC – other cost; TLC – total labour costs; BOLR – basic operator labour rate; g MP – gram of main product.
*calculated based on benefits, operating supplies, supervision cost and administration cost.

The purchase price of CleanCap 5' capping analogues at GMP grade was received from the supplier, TriLink BioTechnologies Inc [11]. The purchase price of the modified UTP (N1-methylpseudouridine-5'-triphosphate) was estimated based on the selling price of this material taking into account a discounting factor obtained by dividing the list price of the CleanCap AU 5' capping analogues with the price quoted by TriLink BioTechnologies Inc for large scale GMP grade supply of the same material. Subsequently, a purchase price value was also received for the modified UTP from TriLink BioTechnologies Inc which was within the uncertainty range listed in **Figure 1**.

The SuperPro Designer modelling files and data is available in a publicly accessible repository: https://github.com/ZKis-ZK/LNP-formulated-RNA-vaccine-drug-substance-production-cost-modelling

References:

1. Rommelag AG. Blow-Fill-Seal Solutions [Internet]. Waiblingen, Germany: Rommelag Kunststoff-Maschinen Vertriebsgesellschaft mbH; 2017. Available from: https://www.rommelag.com/fileadmin/user_upload/Files/CMO/Downloads/EN/Rommelag-Engineering-Products-Brochure-CMO-EN.pdf

2. Rommelag AG. Rommelag bottelpack bp460 - Aseptic filling in ampoules [Internet]. YouTube. 2017 [cited 2020 Jun 12]. Available from: https://www.youtube.com/watch?v=djYqnMipKS8

3. MEDInstill. INTACT™ Modular Filler (IMF) [Internet]. 2020 [cited 2020 Apr 20]. Available from: http://www.medinstill.com/intact_modular_filler_imf.php

4. Kis Z, Kontoravdi C, Dey AK, Shattock R, Shah N. Rapid development and deployment of high-volume vaccines for pandemic response. J Adv Manuf Process [Internet]. 2020/06/29. John Wiley & Sons, Inc.; 2020 Jul;2(3):e10060. Available from: https://www.ncbi.nlm.nih.gov/pmc/articles/PMC7361221/

5. Bancel S, Issa, William J, Aunins, John G, Chakraborty T. Manufacturing methods for production of RNA transcripts [Internet]. USA: United States Patent and Trademark Office; WO/2014/152027; PCT/US2014/026835; US20160024547A1, 2014. Available from: https://patentimages.storage.googleapis.com/7a/bb/8f/5ce58cdaa18a0d/US20160024547A1.pdf (accessed on 10.Nov.2020)

6. Scorza Francesco Berlanda, Yingxia Wen, Andrew Geall, Frederick Porter. RNA purification methods [Internet]. 20160024139, EP2970948A1; WO2014140211A1, 2016 [cited 2018 May 1]. Available from: https://patents.google.com/patent/EP2970948A1/no

7. Heartlein M, Derosa F, Dias A, Karve S. Methods for purification of messenger rna [Internet]. USA; DK14714150.1T; PCT/US2014/028441, 2014. Available from: https://patents.google.com/patent/DK2970955T3/en (accessed on 15.Dec.2019)

8. Funkner A, Dorner S, Sewing S, Kamm J, Broghammer N, Ketterer T, et al. A method for producing and purifying rna, comprising at least one step of tangential flow filtration [Internet]. Germany: World Intellectual Property Organization; PCT/EP2016/062152; WO/2016/193206, 2016. Available from: https://patentscope.wipo.int/search/en/detail.jsf?docId=WO2016193206 (accessed on 10.Oct.2020)

9. Kis Z, Shattock R, Shah N, Kontoravdi C. Emerging Technologies for Low-Cost, Rapid Vaccine Manufacture. Biotechnol J [Internet]. John Wiley & Sons, Ltd; 2019 Jan 1;14(1):1800376. Available from: https://doi.org/10.1002/biot.201800376

10. Centre for Process Innovation Limited. Telephone and email correspondence with biopharmaceutical manufacturing experts from the Centre for Process Innovation Limited, UK - Jul 2018. Darlington, UK: CPI; 2020.

11. TriLink. Telephone conversation with representatives from TriLink, Inc. on 10 April 2020. San Diego, CA, USA: TriLink; 2020.
